# The N-Terminal Amphipathic Helix of the Topological Specificity Factor MinE Is Associated with Shaping Membrane Curvature

**DOI:** 10.1371/journal.pone.0021425

**Published:** 2011-06-27

**Authors:** Yu-Ling Shih, Kai-Fa Huang, Hsin-Mei Lai, Jiahn-Haur Liao, Chai-Siah Lee, Chiao-Min Chang, Huey-Ming Mak, Cheng-Wei Hsieh, Chu-Chi Lin

**Affiliations:** 1 Institute of Biological Chemistry, Academia Sinica, Taipei, Taiwan; 2 Institute of Biochemical Sciences, National Taiwan University, Taipei, Taiwan; Universite Libre de Bruxelles, Belgium

## Abstract

Pole-to-pole oscillations of the Min proteins in *Escherichia coli* are required for the proper placement of the division septum. Direct interaction of MinE with the cell membrane is critical for the dynamic behavior of the Min system. In vitro, this MinE-membrane interaction led to membrane deformation; however, the underlying mechanism remained unclear. Here we report that MinE-induced membrane deformation involves the formation of an amphipathic helix of MinE^2–9^, which, together with the adjacent basic residues, function as membrane anchors. Biochemical evidence suggested that the membrane association induces formation of the helix, with the helical face, consisting of A2, L3, and F6, inserted into the membrane. Insertion of this helix into the cell membrane can influence local membrane curvature and lead to drastic changes in membrane topology. Accordingly, MinE showed characteristic features of protein-induced membrane tubulation and lipid clustering in in vitro reconstituted systems. In conclusion, MinE shares common protein signatures with a group of membrane trafficking proteins in eukaryotic cells. These MinE signatures appear to affect membrane curvature.

## Introduction

Targeting of proteins to specific destinations at the appropriate time is crucial for cell function. This process often involves specific protein motifs, and requires the intricate regulation and coordination of different cellular components. Protein targeting is involved in prokaryotic cell division, during which a series of proteins are assembled in a hierarchical order to form a division septum at the correct mid-cell position. An essential component of the division apparatus is the tubulin homolog FtsZ; this is precisely located at the midpoint of the cell, where it forms a ring-like structure underneath the membrane and recruits other division proteins (reviewed in [1]). In *Escherichia coli* (*Ec*), the position of the FtsZ ring is regulated by the Min system [2], which is composed of three proteins, MinC, MinD, and MinE; these cooperate to form a dynamic oscillator that guides the placement of the FtsZ assembly. MinC is a negative regulator of the FtsZ ring [3], [4], and MinD associates with the cell membrane and undergoes a pole-to-pole oscillatory localization cycle in the presence of MinE and ATP [5], [6]. The Min system is a simple but dynamic and functional unit that has received attention from researchers involved in a variety of scientific disciplines [7], [8], [9]. However, the underlying mechanisms responsible for the membrane-association properties of the Min system require further investigation.

Correct functioning of the Min system involves the formation of membrane-associated polymeric structures of MinD [10], [11], [12]. MinD accumulates in the membrane at a polar zone at one end of the cell. It associates with the cell membrane as a MinD-ATP complex through its C-terminal amino acids, which fold into an amphipathic helix [13], [14]. Upon membrane association, MinD polymerizes into a tightly coiled helix extending from the originating pole almost to the midpoint of the cell [15]. MinE forms a ring-like structure at the mid-cell and stimulates MinD's ATPase activity. This drives its release from the membrane and causes retraction of the leading edge of the MinD polar zone back towards the pole [12]. Recently, we demonstrated that MinE is capable of associating with the cell membrane through its N-terminal domain [16]. A mutant MinE containing residue substitutions at positions R10, K11, and K12 was deficient in membrane binding and unable to support normal MinD/E localization and oscillation cycles; however, MinE's ability to stimulate MinD ATPase activity was unaffected. This suggests that direct MinE interaction with membranes is critical for the functioning of the Min system, and that stimulation of the MinD ATPase activity alone is not sufficient. Interestingly, under a transmission electron microscope, purified MinE caused phospholipid vesicles reconstituted from *E. coli* lipids to deform into tubules that were surrounded with a discrete coat. These data indicate that MinE can induce membrane deformation, change membrane topology, and provide a physical force. This force may act with ATP hydrolysis in MinD to remove MinD molecules from membranes during the disassembly stage of the oscillation cycle [16].

Examples of protein-induced membrane deformation in prokaryotes are limited. MinD is known to form arrays of helical filaments surrounding membrane tubules [10], but the function of this phenomenon is not fully understood. It was proposed that the dynamics of the FtsZ ring generate a force that constricts the membrane at the division site [17]. *In vitro* evidence also suggests that the constriction force of the FtsZ ring is caused by filament bending. The intrinsic curvature of FtsZ protofilaments is known to generate bulges and convex depressions in membranes and to deform liposomes following fusion with the amphipathic helix of MinD [18]. The bacterial dynamin-like protein (BDLP) of *Nostoc punctiforme* showed helical self-assembly and tubulation of a lipid bilayer *in vitro*, which may represent a transitional stage of BDLP-mediated membrane fission and fusion [19], [20]. MinD and BDLP share common features of self-assembly on the membrane and nucleotide-mediated conformational changes; however, BDLP is anchored to the membrane by a hydrophobic paddle, while MinD is attached by an amphipathic helix.

In this work, we have identified an additional functional motif of MinE that is associated with MinE-induced membrane deformation. We have provided direct evidence that the extreme N-terminus of MinE from *E. coli* folds into an amphipathic α-helix when associated with a membrane. This property differed from MinE from *Neisseria gonorrhoeae* (*Ng*), which showed a stable N-terminal helix in solution [21]. Meanwhile, we have further monitored MinE-induced membrane deformation using *in vitro* systems of synthetic giant liposomes and supported lipid bilayers (SLBs) via time-lapse fluorescence microscopy. This MinE-induced membrane deformation required both the earlier identified charged residues R10, K11, and K12 [16] and the amphipathic motif identified in this report. Disturbing the amphipathicity in this region not only led to failure to deform the membrane *in vitro*, but also caused alterations in protein stability, which may serve as a control mechanism for the regulation of the cellular concentration of MinE. In summary, this study of MinE illustrates the universal mechanisms involved in the targeting of peripheral membrane proteins that are capable of causing membrane deformation; such mechanisms have prokaryotic and eukaryotic origins.

## Results

### MinE^2-9^ inserts into the membranes as an amphipathic helix

**Figure 1:**
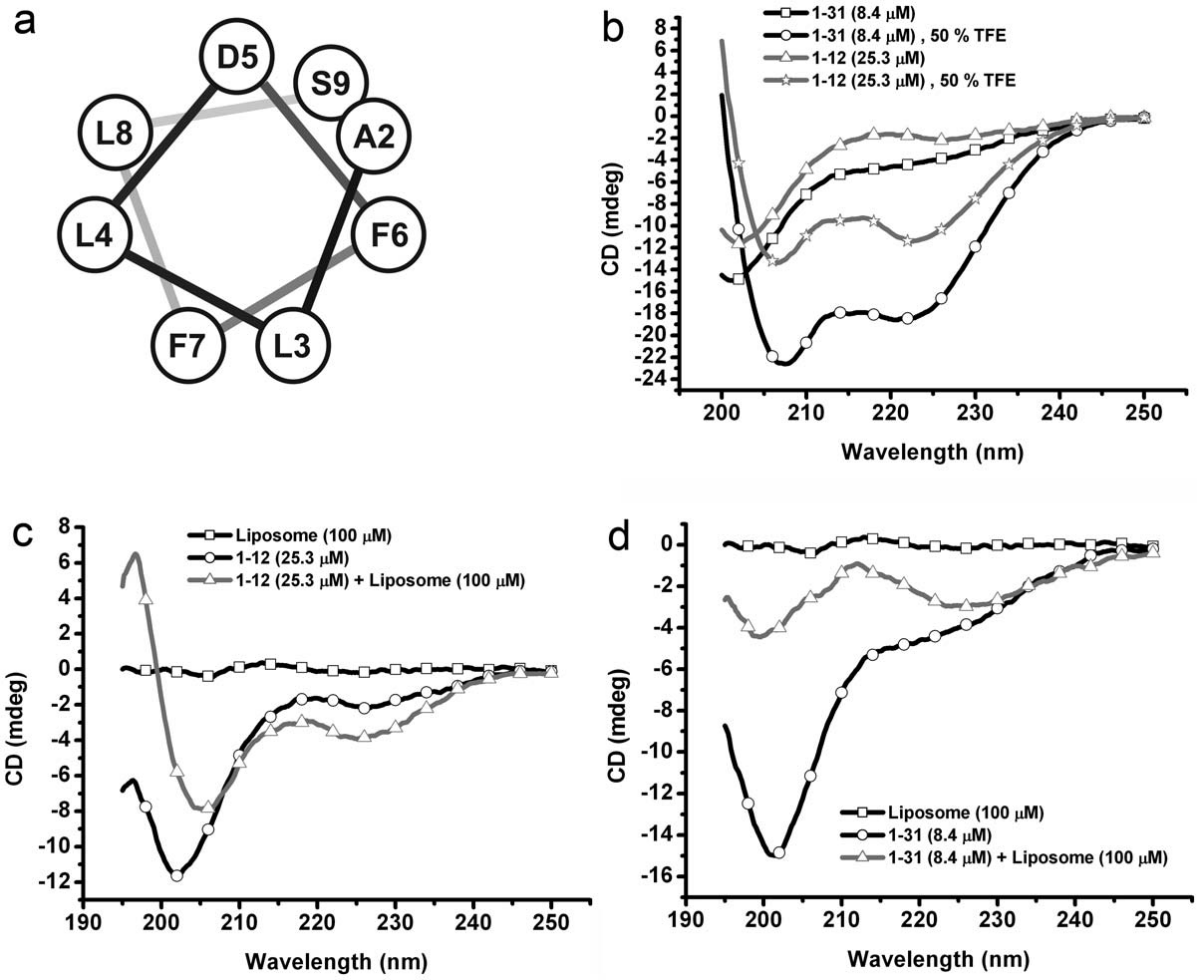
MinE^2–9^ inserts into the membrane in a helical conformation. (**a**) Helical wheel projection of MinE^2–9^. (**b**) The propensity of MinE^1–31^ and MinE^1–12^ for helical folding, measured using circular dichroism, in the presence or absence of 50% trifluoroethanol (TFE). (**c**) The propensity of MinE^1–12^ for helical folding, measured using circular dichroism, in the presence or absence of 100 µM liposomes. (**d**) The conformational changes of MinE^1-31^, measured using circular dichroism, in the presence or absence of 100 µM liposomes.

To investigate whether other mechanisms besides the electrostatic interaction are involved in mediating the MinE-induced membrane deformation, we analyzed the MinE protein sequence using helical wheel projection programs. We found that residues 2–9 were capable of forming an amphipathic helix of 1–2 helical turns (Figure 1a). Residues A2, L3, L4, F6, F7, and L8 formed a large non-polar, hydrophobic face, and residues D5 and S9 were located on a hydrophilic surface. The extreme N-terminus of MinE from 11 other bacterial species showed propensities to form amphipathic helices, and had 4–6 residues located on a hydrophobic surface (Figure S1). The high conservation of amphipathic helix formation was suggestive of its importance, and led us to hypothesize that this amphipathic helix, along with the basic residues R10, K11, and K12 [16], served as a membrane anchor that sustains the peripheral association of MinE.

To explore this hypothesis, we took advantage of the characteristic spectral shift of tryptophan fluorescence emission that occurs as a function of solvent polarity and serves as a measure of peptide-membrane interactions [22]. A single tryptophan substitution was introduced in MinE^1–31^ during peptide synthesis to replace residues A2, L3, L4, F6, F7, or L8. A tryptophan residue added to the C-terminus of MinE^1-31^ served as a control. The amount of liposome supplied in the experiments was reduced to 10 µM to minimize scattering interference. To mimic a cardiolipin-enriched membrane, we used liposomes made of PE:PG:CL  = 36:14:50 mol%. A significant blue shift of the maximal emission wavelength was recorded for the A2W (10.66±2.61 nm), L3W (13.33±1.34 nm), and F6W (7.17±1.71 nm) substitutions (Table 1; Figure S2). This was higher than the peptides bearing L4W (4.78±1.57 nm), F7W (4.00±0.88 nm), L8W (4.44±0.20 nm), and W32 (3.56±1.36 nm) substitutions. Interestingly, peptide MinE^1–12^ with the F6W substitution showed a mild blue shift (2±0.77 nm), indicating an indispensible role for residues 13–31 in stabilizing the peptide-membrane interaction. Taken together, the results suggest that the helical face of MinE, consisting of A2, L3, and F6, forms a hydrophobic surface that is oriented to interact with the hydrophobic regions of the phospholipid bilayer.

**Table 1:** Summary of the tryptophan blue shift assays of the MinE peptides.

Peptide		Blue shift (nm)
1–31	A2W	10.66±2.61
	L3W	13.33±1.34
	L4W	4.78±1.57
	F6W	7.17±1.71
	F7W	4.00±0.88
	L8W	4.44±0.20
	W32	3.56±1.36
1–12	F6W	2.00±0.77

### A helical conformation of MinE^2-9^ is induced upon association with the membrane

To further investigate the helix forming ability of MinE and its association with the membrane, we measured the far-UV circular dichroism (CD) spectra of MinE^1-12^ and MinE^1-31^ in the presence or absence of liposomes (PE:PG:CL = 36:14:50 mol%; Figure 1b–d). Interestingly, MinE^1–12^ and MinE^1–31^ in buffer may have adopted a polyproline II (P_II_)-like conformation, as suggested by strong negative values near 200 nm and elevated readings at 220 nm in the spectra (Figure 1b). The P_II_ conformation is a left-handed threefold helix of nominally unordered peptides in their charged forms. By the addition of 50% trifluoroethanol (TFE), which is known to stabilize the helical structures of proteins and peptides, spectra of both MinE^1–12^ and MinE^1–31^ showed characteristic features of a high helical content, i.e. the troughs around 208 and 222 nm (Figure 1b). MinE^1-12^ showed typical features of high helical contents when 100 µM liposomes were added in the reaction (Figure 1c). This further expanded a previous theory that a nascent helix of MinE^1-22^ in solution [23] may be stabilized by interacting with the cell membrane. We also detected significant changes in the CD spectrum of MinE^1-31^ with liposomes (Figure 1d), but the overall secondary structure was more complicated. Part of the reason may be because of aggregation of the peptide when associated with the liposomes [16], as indicated by reduction of the signal. In summary, our results suggest that the extreme N-terminal region of MinE has a strong propensity to fold into a helix during membrane association.

### Molecular dynamics simulation of interactions between MinE^2-12^ and membranes

**Figure 2:**
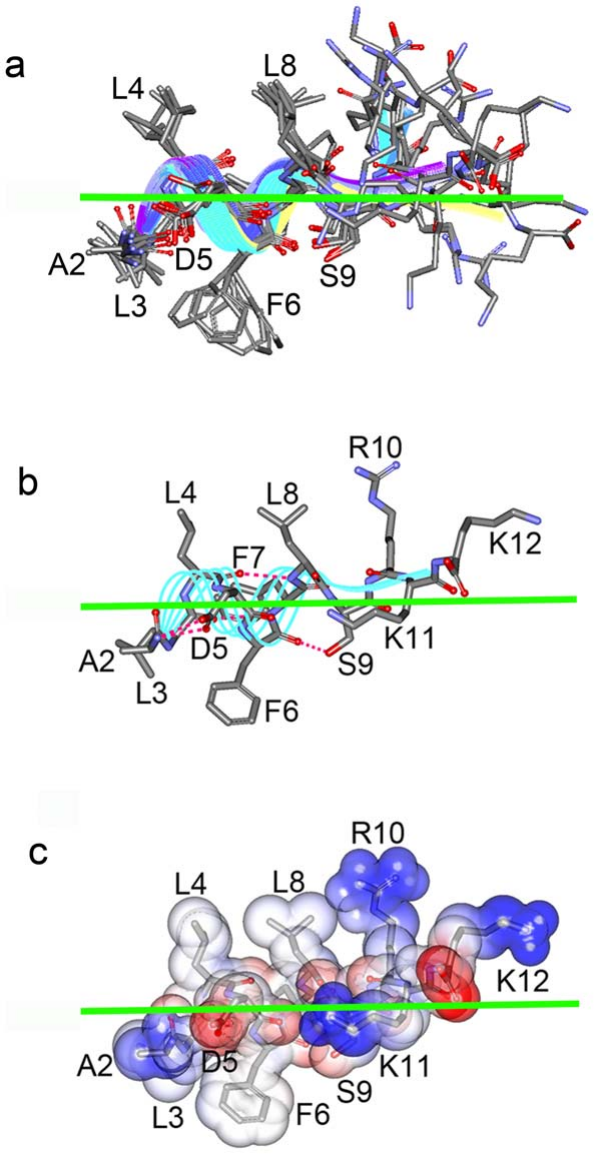
Molecular dynamics simulation of the MinE^2–12^–membrane complex. (**a**) Superimposition of selected intermediates over a 10 ns simulation (starting model, 1 ps, 2.5 ns, 5 ns, 7.5 ns, and 10 ns). The parallel color lines represent the helical conformations of the intermediates. (**b**) The final conformation after a 10 ns simulation. The parallel cyan lines represent the helical conformation. Magenta dash line: hydrogen bond. (**c**) Charge-potential surface of the final conformation of the peptide-membrane complex. The charge potential from positive to negative is colored from blue to red. Green horizontal line: membrane boundary.

In addition, we used the molecular dynamics simulation to model how MinE^2-12^ was positioned in the membrane (Figure 2, S3). We studied MinE^2–12^ because the first methionine residue of MinE was cleaved off in *E. coli* [16]. The starting model of MinE^2–12^ was constructed based on the NMR structure of *Ng*MinE^2-12^, in which residues 2–8 showed an α-helical conformation and the rest of residues are in a loop region [21]. The procedure of adding a virtual membrane of 30 Å thickness generated a model of the peptide sitting at the interface region of the membrane. Information from the tryptophan blue shift assays allowed us to manually adjust the orientation of the MinE^2–12^ molecule so that the side chains of A2, L3, and F6 were positioned in the membrane in the initial model. The side chains of D5, S9, and R10 were also positioned in the membrane through this operation (Figure S3a: starting model). This peptide-membrane complex was then simulated using an implicit solvent model, as suggested for studying the peptide-membrane association [24], [25].

The conformation trajectory of a 10 ns simulation (Figure 2, S3) suggested that the major conformational changes occurred in the loop region, where the side chains of R10 and K11 were repositioned out and in the membrane, respectively (Figure 2a, S3a). The side chains of residues 2–8 showed constant locomotion because of their interactions with the membrane environment, but their relative orientations to the membrane were unchanged. The charge coming from the side chain of D5 was neutralized by the formation of a salt bridge with the N-terminal amino group of A2. The conformation trajectories also suggested that the interface localization of MinE^2–12^ was maintained by hydrophobic interactions between side chains of A2, L3, and F6 and the membrane (Figure 2, S3a). The benzyl group of F6 appeared to insert deeper into the phospholipid bilayer. The presence of side chains of D5, S9, and K11 in the membrane may be explained by polar interactions with the head groups of the bilayer. This simulation provided a specific view of the folding and positioning of MinE^2-12^ when associated with the membrane. It should be noted that the simulation process did not account for the bending flexibility of the membrane; in reality, insertion of such a helix into a membrane is likely to induce bending [26].

### MinE induced liposome deformation in real-time

**Figure 3:**
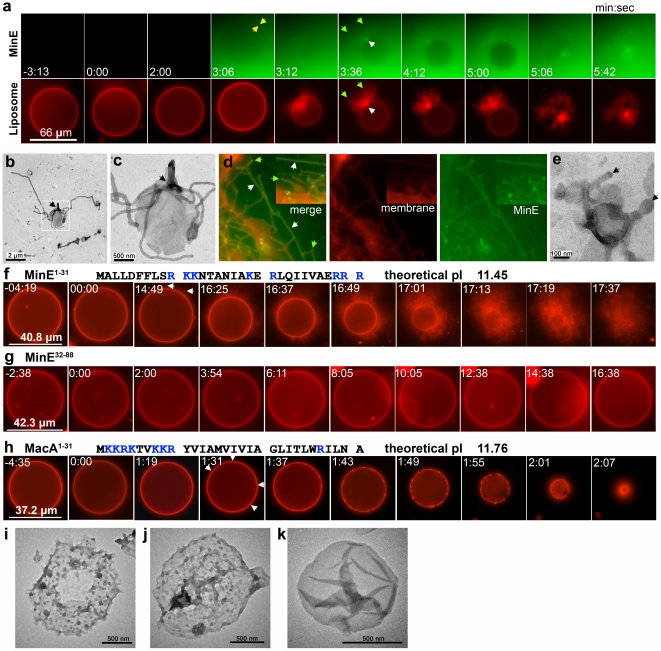
MinE induced liposome deformation in vitro. (**a**) A time-lapse sequence of liposome deformation caused by full-length MinE. Time zero was defined as the first frame acquired after the addition of the protein. Massive tubules burst out from a confined area on a liposome (white arrow), which was subsequently deformed into massive tubules. Alexa Fluor 488-labeled MinE was visible around the periphery of the liposome (yellow arrows) and at the place where tubules emerged from the liposome; MinE colocalized with the membrane tubules (green arrows). (**b,c**) TEM images of MinE-induced tubule formation at one position on a liposome. Micrograph (c) is the same as the boxed area in (b), taken under higher magnification. The arrows indicate the position at which the membrane tubules emerged. (**d**) Colocalization of MinE with membrane tubules under the fluorescence microscope. The white arrows indicate colocalized areas of MinE and membrane tubules. The green arrows show the tips of tubules where concentrated fluorescent MinE appeared. (**e**) A TEM image of liposome budding. Arrows show the electron-dense caps on the buds. (**f**) MinE^1–31^ induced the emergence of membrane tubules from the entire periphery of a liposome, followed by complete deformation. Arrows indicate peripheral membrane tubules. (**g**) A control experiment using MinE^32-88^ in the liposome deformation assay. (**h**) Time-lapse sequence of liposome deformation caused by MacA^1–31^. Arrows show the formation of clusters containing Texas Red-DHPE at the periphery of a liposome. Primary sequences and theoretical pI values of MinE^1–31^ and MacA^1–31^ are presented above each image series. Basic residues are presented in gray. The scale bar represents the diameter of the liposome in (a), (f–h). (**i–k**) Electron micrographs of liposome deformation caused by MacA^1–31^ (i, j) and a control liposome (k).

MinE was found to induce liposome deformation in association with direct MinE-membrane interactions [16]. To better characterize this deformation process, and establish the correlation between insertion of an amphipathic helix and membrane deformation, we set up an imaging system to simultaneously visualize the protein-liposome interaction using a wide-field fluorescence microscope. We used Alexa Fluor 488-labeled MinE and liposomes (PE:PG:CL  = 65:25:10 mol%) doped with 0.2 mol% Texas Red-DHPE for visualization (Figure 3a, S4a). In each time-lapse sequence acquisition, we imaged an isolated liposome for a short period of time before addition of the protein. In reactions with wild-type MinE, the periphery of the spherical liposomes gradually became coated with green fluorescence (Figure 3a; yellow arrow). Moments later, the liposomes either suddenly burst or gradually deformed into membrane tubules from a confined area (Figure 3a; white arrow). Membrane tubules emanating from a liposome were also observed with an electron microscope (Figure 3b,c). Fluorescent MinE was colocalized with the membrane tubules (Figure 3a; green arrow), indicating that tubule formation is associated with MinE.

We also examined reactions that were incubated for 10 min prior to mounting on clean glass slides. In these experiments, adsorption of lipids to the glass surface simplified the imaging process. MinE colocalized with various parts of the lipid tubules and liposomes (Figure 3d; white arrow), or concentrated at the tips of the tubules (Figure 3d; green arrow). This is consistent with electron microscope observations, which showed electron dense caps on buds sprouting from liposomes (Figure 3e; arrows). Membrane deformation and tubulation have been associated with some membrane trafficking proteins in eukaryotes (Table S1); here, we demonstrate that a prokaryotic protein has the same activity.

### MinE^1–31^ is fully capable of inducing membrane tubule formation

Interestingly, we found that N-terminal MinE^1–31^ was able to induce membrane deformation of the giant liposomes in our experimental setup (Figure 3f, S4b). The initiating points of the deformation process were significantly different for the wild-type protein and MinE^1–31^. The full-length protein induced liposome deformation at a focal point (Figure 3a; white arrow); MinE^1–31^ initiated tubule formation around the entire periphery of the liposome (Figure 3f). These data suggest that the C-terminal domain of MinE is required for localizing the deformation activity to a specific area of the membrane environment. This may involve dimerization [27] or a higher-order pattern of organization of the C-terminal domain [28].

The control experiment showed that no membrane deformation occurred with the addition of MinE^32–88^ (Figure 3g, S4c); this supports the conclusion that the formation of membrane tubules is an intrinsic property of MinE^1-31^. Interestingly, the localized tubulation induced by MinE^1-31^ was not fully restored by adding the C-terminal MinE^32-88^ *in trans* (Figure S5), indicating that the N- and C-terminal domains as an integral whole are necessary for conformation and function. The N-terminal domain of MacA, a component of the macrolide-specific ABC-type efflux carrier of *E. coli* strain APEC 01, was used as another control in the time-lapse liposome deformation experiments (Figure 3h, S4d). MacA^1–31^ shares common features with MinE^1–31^ in its primary sequence, but not in the organization of the charged and hydrophobic residues. The first 10 residues of MacA are positively charged and thought to be a signal peptide; the amino acids following the signal peptide are enriched in hydrophobic residues. MacA^1–31^ induced clustering of fluorescent lipids on the periphery of the liposomes (Figure 3h, arrows), and subsequently caused them to shrink; there were no identifiable protrusions indicating tubulation. Under the electron microscope, MacA^1–31^ induced granulation and became poriferous on liposomes (Figure 3i,j). This was in clear contrast to MinE-induced membrane tubule formation and the smooth surface of the liposome alone (Figure 3k). Results from both fluorescence and electron microscopy approaches suggested that membrane-tubulating activity is an intrinsic function of MinE^1–31^.

### MinE-induced deformation of the supported lipid bilayers

**Figure 4:**
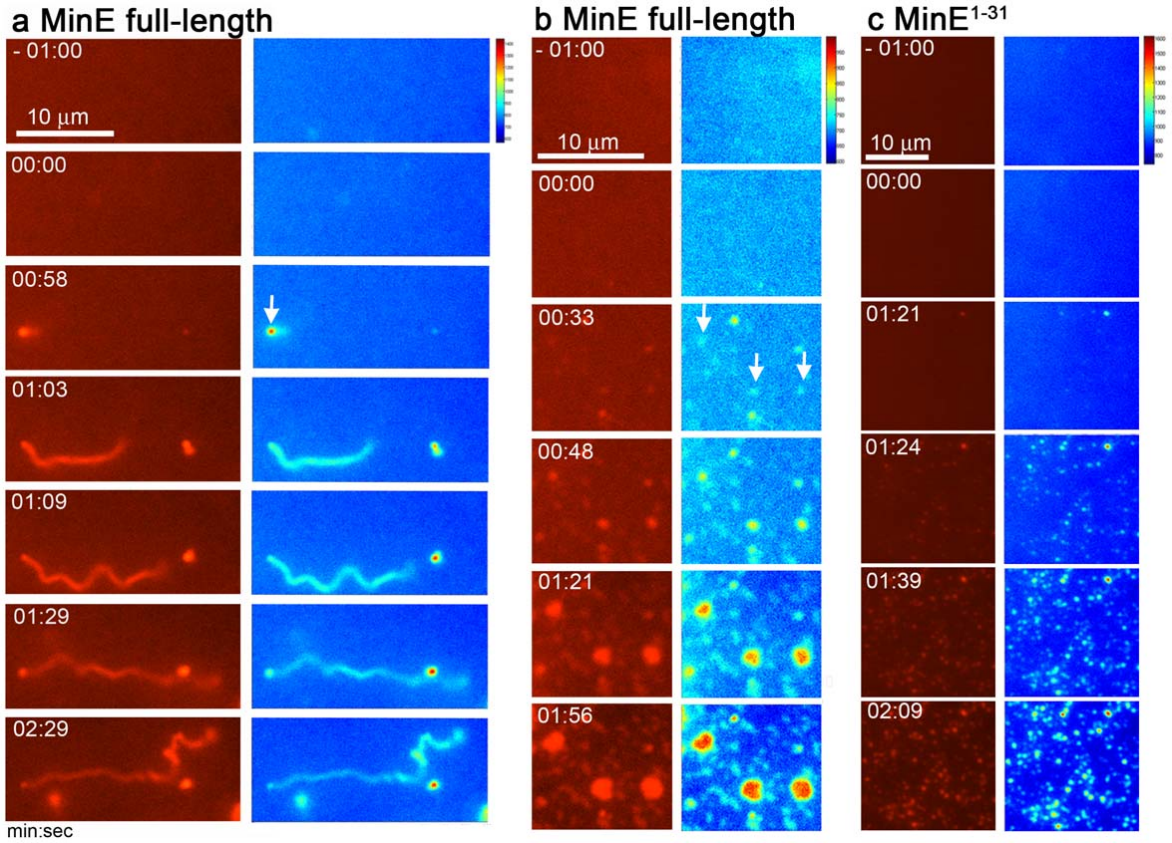
Full-length MinE and MinE^1–31^ induced deformation of fluorescently labeled SLBs. (**a**) MinE induced membrane tubule formation from SLBs. (**b,c**) Patchy fluorescence accumulation on SLBs caused by MinE (b) and MinE^1–31^ (c). Note also the drastic reduction of fluorescence outside the fluorescent patches. In each image set, the left column contains original micrographs and the right column contains the corresponding fluorescence intensity maps. Time zero was defined as the first frame acquired after the addition of the protein. The occasional brighter objects in the images were caused by impurities floating through the imaged fields. Arrows indicate the initiation points of tubule and patch formation.

We further examined MinE-induced membrane deformation using supported lipid bilayers (SLBs) prepared with *E. coli* polar lipids (PE:PG:CL  = 65:25:10 mol%; Figure 4). The fluidity of the bilayer was demonstrated to show its functionality under our experimental conditions (Figure S6). Before addition of the protein we identified an area on the labeled SLBs that showed even distribution of fluorescence, reasonable background fluctuation over time, and limited bright and broken spots. MinE was applied in solution and allowed to diffuse over the SLBs; this induced the accumulation of bright fluorescent foci that sometimes accompanied a significant reduction in background fluorescence (Figure 4a,b). As time progressed, some fluorescent foci remained unchanged, but the intensity of others increased as they developed into tubules (Figure 4a) or spread laterally to form larger fluorescent patches (Figure 4b). These patches may have originated as membrane tubules laid down on the mica surface during image acquisition, and subsequent enlargement might have been due to the diffusion of phospholipids from accumulation sites. Membrane tubules induced by MinE were coiled and bent (Figure S7a), this differed from the smooth contour of those caused by the external force of buffer purposely blown over the SLBs (Figure S7b). The images of fluorescently labeled MinE colocalized with membrane tubules indicated that tubule formation was associated with MinE (Figure S7a).

Replacing wild-type MinE with MinE^1–31^ in the SLB experiments resulted in the formation of fluorescent foci, but no obvious membrane tubules were seen (Figure 4c, S7c,d). Atto488-labeled anti-MinE antibody was used to identify MinE^1–31^ on the fluorescent patches. MinE^1–31^ was found at the vicinity of the lipid patches, but was not completely superimposed on them (Figure S7c; cyan arrow). We also identified arcs (Figure S7c; yellow arrow) and enclosed rings (Figure S7c; white arrows) of MinE^1–31^ surrounding larger lipid patches. These data suggest that the association of MinE^1–31^ with membranes resulted in the local accumulation of surrounding phospholipids. The number of phospholipids between the accumulation points significantly decreased and contributed to the reduction in background fluorescence (Figure 4b,c). The differences between MinE^1–31^ induced membrane deformation of giant vesicles and SLBs may reside in the continuity of the lipid supplies. Lipids were continuously drawn into the growing tubules in the giant vesicles until transformation was complete. The initiation points for tubule formation on SLBs were scattered and lipids were drawn independently into separate foci. This resulted in a shortage of lipids, which was not able to support tubule growth. These data indicate that MinE is able to cause membrane deformation and induce tubule formation in a flat membrane, which further confirms our observation using the giant liposome system.

### Importance of MinE^1–12^ in membrane association and protein stability

**Figure 5:**
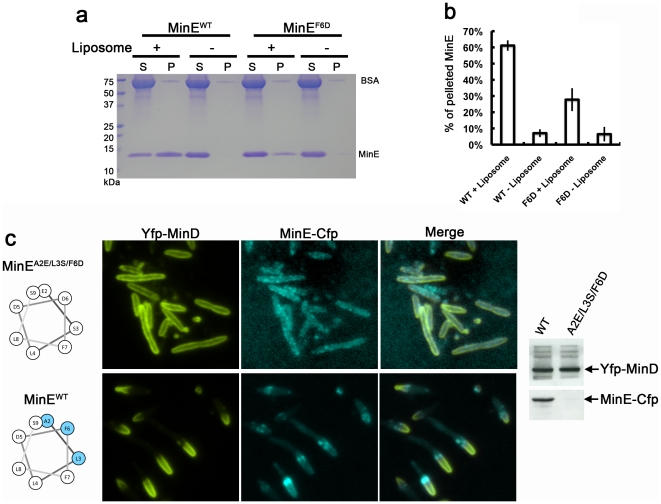
Mutations in MinE^2–9^ affected lipid binding in vitro and protein stability in vivo. (**a**) Full-length MinE carrying a single residue substitution (F6D) to weaken the amphipathicity of MinE^2–9^ showed reduced co-sedimentation with liposomes. S, supernatant; P, pellet. (**b**) Statistical analysis of the ability of MinE^F6D^ to co-sediment with liposomes. Three experiments were used to quantify the supernatant and pellet fractions for analysis. (**c**) Cellular localization of Yfp-MinD, MinE^A2E/L3S/F6D^-Cfp, and wild-type MinE-Cfp. Western blot analyses showed that the mutant protein MinE^A2E/L3S/F6D^ was unstable in cells (right panel) and influenced MinDE localization (left panel). Membrane association through the correct folding of MinE^2–9^ may be critical for MinE protein stability in cells. Monoclonal anti-GFP antibody (Santa Cruz Biotechnology, sc-9996) was used for detecting the fusion proteins.

We constructed a mutant MinE protein by substituting F6 with aspartic acid to weaken the amphipathicity of MinE^2–9^. In the sedimentation assays, the purified mutant protein MinE^F6D^ only retained 45% of the ability to co-sediment with liposomes (PE:PG:CL = 36:14:50 mol%; Figure 5a,b), indicating the importance of this residue in supporting the protein-membrane interaction. The remaining hydrophobic residues, A2 and L3, and the charged residues R10, K11, and K12 may have sustained part of the interaction. In addition, the large hydrophobic face might have allowed the mutant helix to rotate and associate with the membrane. Time-lapse fluorescence microscopy was used to examine liposome deformation induced by the mutant MinE proteins C1 (R10G/K11E/K12E) and MinE^F6D^, which were defective in membrane association. We acquired images for a minimum of 20 min for each experiment and observed five liposomes > 15 µm in diameter for each mutant protein. All five liposomes studied for wild-type MinE showed complete (4/5) or partial (1/5) deformation (Figure S4a); the partially deformed liposomes were likely to progress to full deformation. There was no liposome deformation with the C1 and MinE^F6D^ mutant proteins (Figure S8a,b). Interestingly, although MinE^F6D^ retained approximately half of the membrane binding activity in the sedimentation assay (composition of liposomes PE:PG:CL = 36:14:50 mol%), it failed to bind and deform liposomes (PE:PG:CL = 65:25:10 mol%) under the fluorescence microscope (Figure S8b,c). We conclude that the C1 and MinE^F6D^ mutant proteins are defective in both membrane-association and liposome deformation.

The pSOT169 (P*_lac_*-*yfp::minD minE^A2E/L3S/F6D^::cfp*) construct was generated to further investigate the physiological relevance of the extreme N-terminal helix. The triple mutant was created because the single substitution mutant F6D still retained approximately half of its membrane association ability, even though it failed to deform liposomes. This resulted in no significant changes in MinDE localization when the mutant MinE^F6D^ was expressed in cells. The defect detected in the sedimentation assay may be overcome by the complexity of the cellular environment, including MinD's recruitment of MinE to the membrane location and enrichment of cardiolipin at the division site. When the triple mutant MinDE^A2E/L3S/F6D^ expression was induced in a Δ*min* strain YLS1, MinD was delocalized from the polar zone into a peripheral pattern and MinE^A2E/L3S/F6D^ was dispersed or accumulated as punctuates in the cells (Figure 5c). Western blot analysis detected a low abundance of the MinE^A2E/L3S/F6D^-CFP fusion protein in cells, indicating that the mutant protein was unstable. This instability was more severe than that of the C1 mutant, which was stable when fused to CFP, but unstable when expressed alone [16]. Although the results did not allow us to draw an apparent link with cellular localization, they suggest that proper folding of MinE^2–12^ and membrane association may serve as a control mechanism for the regulation of the cellular concentration of MinE, which is critical for sustaining the oscillation cycles of the Min proteins [29].

## Discussion

Amphipathic helices are widely found in proteins participating in membrane-associated biological activities, such as vesicle trafficking, viral fusion, and toxin-induced membrane lysis. The amphipathic nature of the helix serves as a membrane-anchoring motif that locates near the interface region of the cell membrane, often leading to modification of the protein function and the membrane properties.

A generalized mechanism for peripheral membrane association has been proposed [30]. Primary adsorption of a protein onto a membrane is facilitated by non-specific charge interaction and diffusion. This is subsequently stabilized through membrane penetration by protein motifs and binding to specific lipids. Here, we identified the necessary elements in MinE that fulfill this paradigm. In addition to the charged residues R10, K11, and K12 characterized in our previous study [16], MinE^2–9^ has the tendency to fold into an amphipathic helix upon association with a membrane, as determined by the circular dichroism measurements. Tryptophan blue shift assays suggested that the helical face of residues A2, L3, and F6 are positioned in the membrane. The molecular dynamics simulation provided information on the peptide-membrane interaction, which showed specific conformations when it encountered the cell membrane. Meanwhile, deeper insertion of the side chain of F6 may act as a structural landmark to effectively create membrane defects or to target membranes with positive curvature.

To support the importance of the MinE^2–9^ helix for proper function of the Min system, the mutant protein MinE^F6D^ was engineered to weaken the amphipathicity, which significantly reduced the ability of MinE to associate with membranes *in vitro*. A triple mutant MinE^A2E/L3S/F6D^ affected the protein stability *in vivo*. An unbalanced ratio of MinD to MinE resulted in mislocalization of the proteins [29]. Interestingly, an earlier study showed that an N-terminally truncated MinE (MinE^6–88^) retained its ability to suppress division inhibition by MinCD, but still resulted in a minicelling phenotype [31]. This indicates that the extreme N-terminus of MinE is important for the function of the Min system, but does not affect the interaction of MinE with MinD. Therefore, the membrane anchoring mechanism of MinE, including the charge interaction, the formation of an amphipathic helix of MinE^2–9^, and the preference for cardiolipin [16], is independent of the mechanism that regulates the interaction of MinE with MinD.

**Figure 6:**
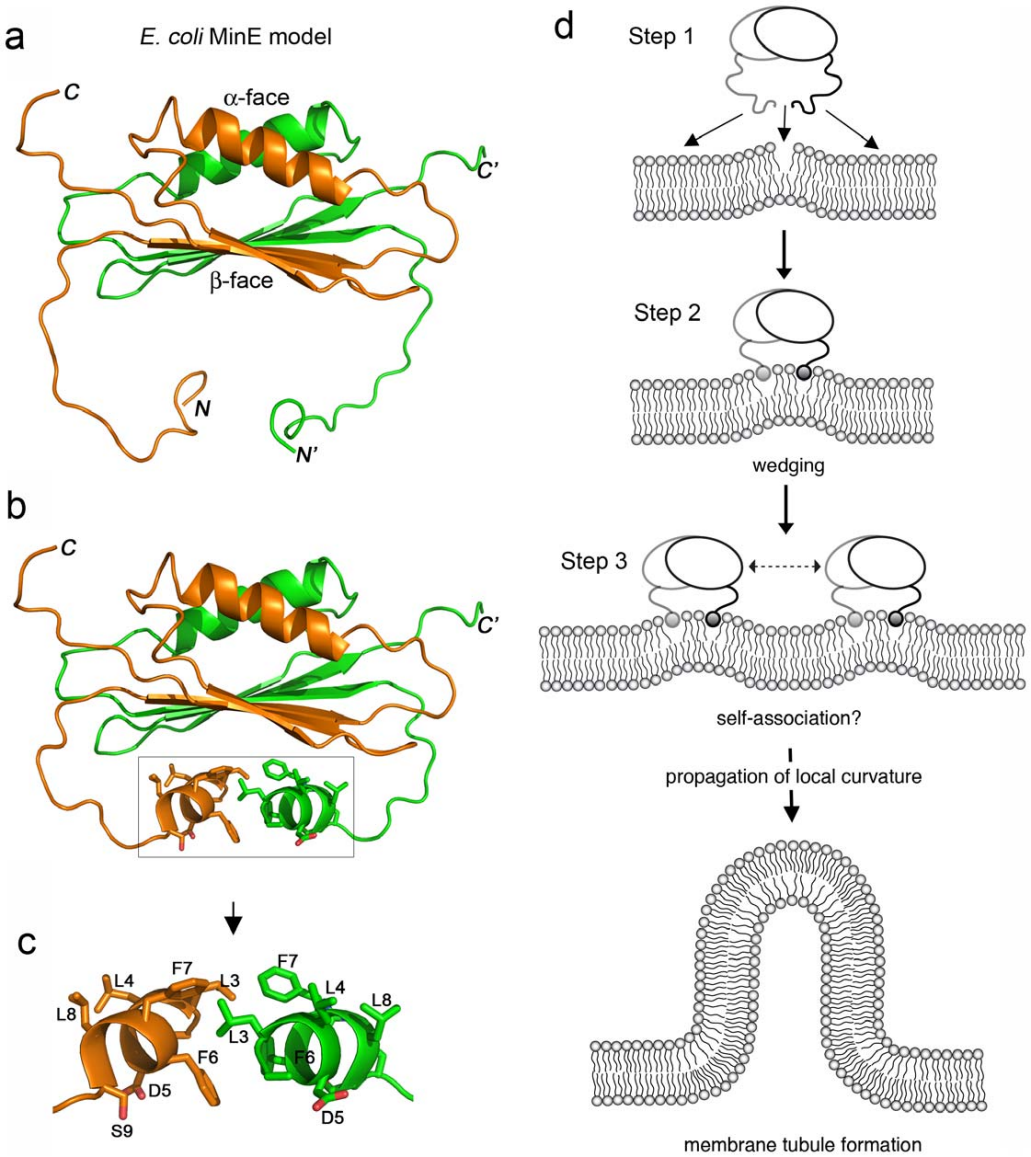
Model of the mechanism underlying MinE-induced membrane deformation. (**a–c**) Predicted dimeric structure of *Ec*MinE that was generated based on the structure of *Ng*MinE (PDB code: 2KXO). Two monomers are colored in orange and green respectively. The extreme N-terminus of *Ec*MinE does not maintain a stable fold (a). When MinE associates with a membrane, the extreme N-terminus of *Ec*MinE folds into an α–helical conformation (b). The orientations of the side chains of this helix are shown in (c). (**d**) A model of the MinE-induced membrane deformation. Step 1: MinE can directly target existing defects on membranes through its N-terminal amphipathic helix (residues 2–9) and the adjacent basic residues (R10, K11, K12). Alternatively, MinE may directly target to a membrane and cause a membrane defect to occur. Both membrane defects and high concentrations of anionic phospholipids will stabilize the initial protein membrane interaction. Step 2: A “wedging effect” on the membranes occurs when an amphipathic helix intercalates into the membranes. Step 3: Localized high density wedges due to self-association of MinE or accumulation of large numbers of wedges can lead to the propagation of membrane defects and more drastic changes in local curvature. This process will overcome an energy barrier and destabilize the membranes, leading to tubule formation.

A recent solved NMR structure of the full-length MinE from *N. gonorrhoeae* showed that the N-terminal helix of residues 2−8 is exposed and connected by an extended loop region to the integral part of the MinE dimer (PDB code: 2KXO) [21]. This structure suggested that the N-terminal amphipathic helix is highly flexible for interactions with other binding partners. Interestingly, the hydrophobic face of the helix, which may be involved in the membrane interaction, was oriented away from the protein surface, suggesting a rotation of the helix is necessary for association with a membrane. Previously, structure determinations of the extreme N-termini of the MinE proteins from *E. coli* and *Helicobacter pylori* were inconclusive [23], [28], which was possibly due to the nature of the MinE proteins from different bacterial species. Our current study demonstrated that the helical conformation of *Ec*MinE^2-9^ was stabilized by the presence of the membrane. Based on the structure information of *Ng*MinE, we modeled the structure of *Ec*MinE for a suggestive view of the N-terminal domain when it forms (Figure 6a−c). In this model, most residues (A2, D5, F6, S9) on the membrane interacting face of the N-terminal helix of *Ec*MinE is exposed on the protein surface and appears accessible for membrane interactions. Therefore, the control mechanism for *Ec*MinE interaction with a membrane may rely on the induced folding property and an interaction between the N-terminal and C-terminal domains to sequester the membrane interaction [16]. Moreover, the similarity of the side chain orientations of residues A2, L3, D5, F6, and S9 in both the molecular dynamics simulation model and the predicted model based on *Ng*MinE, indicated the reliability of the approaches. It will be interesting to see whether targeting the N-terminal domain of MinE to the membrane may trigger conformational changes that expose the MinD interacting sites located on the β-face of the MinE dimer.

The *in vitro* membrane deformation activity of MinE reported in this study, including budding, tubulation, and lipid clustering, is similar to that of several proteins involved in membrane trafficking in eukaryotic cells. Membrane trafficking is a process that allows membranes from different sources to exchange their lipids, proteins, and interior contents. Examples include dynamin, Bar domain proteins (amphiphysin, epsin, endophilin, and nexin), ENTH domain proteins (epsin, Ent3, Ent5), Arf, SarI, Septin, and C2 domain proteins (such as synaptotagmin) (Table S1). Common features shared by these membrane-associating proteins include (1) an amphipathic helix or simply a hydrophobic surface that can insert shallowly into a membrane bilayer, (2) a patch of charged residues that support electrostatic interaction with the membrane, and (3) the unique folding of specific protein domains or a curved shape maintained through self-association to sculpt the cellular membranes [32]–[33] (Table S1). Interestingly, although some of these proteins possess nucleotide triphosphatase activity, there is no evidence, thus far, to demonstrate coupling of nucleotide hydrolysis with induction of membrane tubule formation.

In this study, we present evidence that MinE, the topological specificity determinant of the *E. coli*'s divisome, has membrane deformation activity *in vitro* and possesses signature motifs relating to membrane deformation. By analogy to other membrane curvature sensing and induction mechanisms, we propose a model to explain the involvement of an amphipathic helix in the MinE protein-membrane interaction and MinE-induced membrane deformation. The insertion of the amphipathic helix of MinE into membranes may lead to a local change in curvature that acts as an initiation point for membrane deformation (Figure 6d). This local change in curvature may be propagated through the accumulation of high concentrations of MinE by a process that may or may not involve self-association of MinE, and results in drastic membrane deformation. The enrichment of cardiolipin at the division site of an *E. coli* cell [34], [35] and MinE's higher affinity to anionic phospholipids [16] may in turn contribute to formation of a MinE ring at the midcell, which arrests growth of the MinD polar zone [12]. Both MinE's ability to stimulate MinD ATPase activity and to deform the membrane may contribute to removal of MinD from the membrane location.

In contrast to the list of eukaryotic proteins that possess membrane deformation properties, to our knowledge, MinE, MinD, and BDLP are the only documented prokaryotic proteins that have *in vitro* membrane tubulation activities [10], [16], [20]. These findings indicate that protein-mediated membrane remodeling may occur in bacteria. Although the purpose of such an activity in prokaryotes is not yet fully understood, the activity may contribute to membrane recycling and restructuring during cell growth and development. In plant and animal cells, evidence suggests that membrane trafficking can act as a developmental control during cleavage furrow formation and abscission of daughter cells [36]. Membrane trafficking may involve delivery and sorting of cargo, and deposition of membranes that are linked to the dynamics of the cytoskeleton. The only known equivalent membrane trafficking systems in prokaryotes are an ESCRT-like machinery in wall-less *Crenarchaea* that has been correlated to membrane abscission during cell division [37], [38], and a simple endocytotic system in *Gemmata obscuriglobus* [39]. Further investigations are required to determine whether the protein-induced membrane deformation contributes to effective removal of incorrectly placed septal machinery, and serves as a developmental control in bacteria.

## Materials and Methods

### Tryptophan blue shift assay

The tryptophan blue shift assay was conducted by incubating 6 µM MinE^1–31^ carrying a tryptophan residue at various positions, and 10 µM liposomes (with a diameter of 400 nm) in buffer A (20 mM Tris-Cl, pH 7.5; 200 mM sucrose), at room temperature for 10 min. Control reactions were incubated without liposomes. The mixtures were excited with 280 nm UV light and scanned for fluorescence emission at 300–400 nm on a Fluorolog-3 spectrofluorometer (HORIBA Scientific, Inc.). The statistical value of the blue shift at the maximal emission wavelength was averaged from at least three independent experiments; three continuous scans were repeated in each experiment. We found that the fluorescence intensity gradually decayed during continuous scans of the same sample, thus we did not use fluorescence intensity as an indication of oligomerization.

### Circular dichroism (CD)

The MinE^1–31^ and MinE^1–12^ peptides were dissolved in 20 mM Tris-Cl, pH 7.5 and purified by passing through a 0.22 µm filter and diluted to suitable concentrations before the experiments. Sucrose generates a strong spectral signal at 190 nm; therefore, buffer A was not suitable for this experiment. CD spectra of the peptides were measured in the far UV range (190–250 nm) on a JASCO J-715 spectrometer (JASCO, Japan). The bandwidth and the step resolution were set to 2 nm and 0.2 nm respectively. A quartz cuvette was cleaned by soaking in potassium dichromate solution (10% [w/v] potassium dichromate, 10% [v/v] H_2_SO_4_) and rinsed before use. The optical path of the cuvette was 0.1 cm. For each sample, three scans were performed to obtain an averaged spectrum; this was subtracted from the spectrum of the buffer to provide a baseline correction. When appropriate, 100 µM liposomes were supplied in the reaction.

### Molecular dynamics simulation

The molecular dynamics simulation study was performed using the Discovery Studio 2.5 (Accelrys Inc., San Diego, CA, USA). The starting model of MinE^2–12^ was constructed by replacing the amino-acid side chains of *Ng*MinE^2-12^ (PBD code: 2KXO) with the side chains at *Ec*MinE^2-12^. The CHARMm Polar H force field was applied to the molecule for subsequent simulation. Prior to simulation, a virtual membrane of 30 Å-thickness was added to the molecule using an Implicit Solvent Model GBSW (Generalized Born with a simple SWitching). This step created a model of the *Ec*MinE^2-12^ helix sitting on the interface region of the membrane. The helical face containing A2, L3, and F6 was then manually rotated to face down the membrane, based on the knowledge learned from our experimental data. The resulting model was simulated using the “Standard Dynamics Cascade” protocol consisted of steps of two rounds of minimization, heating, equilibration, and production for 10 ns. During simulation, the backbone carbon atoms of MinE^2-9^ were constrained by a harmonic force with a constant of 1 kcal mole^−1^ Å^−2^. The heating temperature was set between 50 to 300°K. All other settings for the simulation were the same as those for adding a membrane.

### Strains and Plasmids

Strains and procedures for overproduction and purification of MinE, preparation of giant liposomes, and electron microscopy were as previously described [16]. pSOT164 [P*_T7_*::*minE^F6D^-his*] was generated for protein overproduction by introducing a point mutation by a long-range PCR reaction with pSOT13 [16] as the template DNA. pSOT169 [P*_lac_*::*yfp-minD minE^A2E/L3S/F6D^-cfp*] was constructed by subcloning *minE^F6D^* from pSOT164 into pYLS68 [P*_lac_*::*yfp-minD minE-cfp*] [12] followed by a long-range PCR reaction to introduce additional point mutations.

### Fluorescence microscopy

For observing giant liposome deformation, glass slides and cover slips were cleaned by sonication in ddH_2_O, ethanol, acetone, 1 M KOH, and ddH_2_O sequentially for 30 min each, before being soaked in methanol and dried before use. An o-ring was placed on each clean slide to create a sample-holding chamber, and 100 µl 20 mM Tris-Cl, pH 7.5 were added followed by 100 µl of liposome suspension in buffer A. An upright Olympus BX61 microscope equipped with Chroma ET-mCherry and ET-GFP filter sets, a Hamamatsu Orca-AG Cool Charge-Coupled Digital camera, and Volocity (Improvision, PerkinElmer) was used for image acquisition and analysis. A water-immersion objective (Olympus LUMPlanF1 60X/0.9W) was attached to the microscope to view an isolated liposome for a few minutes prior to protein addition. Liposomes were labeled by the addition of 0.2 mol% Texas Red-DHPE (Texas Red 1,2-dihexadecanoyl-*sn*-glycero-3-phosphoethanolamine, Invitrogen), and purified MinE or MinE^F6D^ was labeled using Alexa Fluor® 488 reactive dye with a tetrafluorophenyl (TFP) ester moiety (Invitrogen). The degree of labeling was estimated according to the manufacturer's instructions; every protein molecule was averaged to carry 0.2–0.7 fluorescent dye molecules (mol dye/mol MinE). Liposomes formed in buffer A were diluted two fold in 20 mM Tris-Cl, pH 7.5 in the observation chamber. Protein was added to a final concentration of 6 µM from the side of the chamber. The protein was allowed to diffuse toward the targeted liposome. Simultaneous imaging using both the Gfp and mCherry channels was conducted for at least 20 min or until the liposomes burst. Time zero was defined as the first frame acquired after the addition of the protein. The acquisition interval was fixed as 6 s, unless otherwise specified. The acquired image sequences were processed in Volocity, Matlab, and/or Photoshop for figure presentations. Additional reactions were incubated for 10 min before spotting on the glass slides for single time point observations.

### Preparation of supported lipid bilayers

A total of 0.5 mg/ml *E. coli* polar lipids (Avanti) mixed with 0.4 mol% Texas Red-DHPE in chloroform in a small glass vial were dried under nitrogen and then in a vacuum for an additional 1–2 h. The dried lipid layers were rehydrated in 1 ml buffer A and kept in the dark with intermittent gentle shaking for an hour at room temperature. The liposome suspension was subjected to five to eight freeze-thaw cycles of 1 min in liquid nitrogen and 5 min in water at room temperature. The freezing step fragmented the bilayers, thus enhancing the reformation of unilamellar vesicles in the thawing step. The vesicle suspension was passed through an extruder with filters of pore size 400 and 100 nm, in sequential steps of 21 passages each, to generate small unilamellar vesicles (SUVs) of uniform size. The SUV suspension was diluted 10 fold with buffer A and applied to a chamber with a freshly cleaved piece of mica mounted on a glass slide. After incubation at 37°C for 30 min, 20 mM CaCl_2_ and 100 mM NaCl were added to the chamber to facilitate vesicle fusion and adsorption onto the mica. After incubation at 37°C for an additional 30–60 min, the suspension was carefully drawn out followed by four gentle washes with buffer A. The supported lipid bilayer (SLB) on the mica was immersed in 200 µl buffer A. All studied areas of SLBs were observed for one minute before addition of purified MinE (final concentration, 24 µM) or synthesized MinE^1–31^ peptide (24.5 µM) using the microscopy system described previously. The image sequences were acquired continuously at 3-s intervals without stopping when the proteins were applied. The acquired image sequences were processed as previously described. Fluorescence intensity maps were generated in Matlab; 16-bit images (grey scale range, 0–65535) were analyzed and the color bar was normalized using the lowest and highest intensity values in each image sequence.

### Immunofluorescence detection of MinE^1-31^ on the deformed bilayer

We identified the presence of MinE^1-31^ on the lipid clusters by hybridization. We purified crude anti-MinE antisera by adsorbing anti-MinE antibodies onto purified MinE proteins immobilized on a PVDF membrane by SDS-PAGE separation and western blotting. The adsorbed antibodies were stripped off the membrane in 1 ml pre-chilled 0.2 M glycine, pH 2.5 with gentle shaking for 45 s. The purified antibody solution was immediately neutralized with 1 ml 1 M Tris, pH 9.0 and concentrated to 1.2 mg/ml. This was then conjugated with Atto488 following the manufacturer's instructions [Lightning-Link™ Atto488 Conjugation Kit; Innova Biosciences]. The Atto488-conjugated antibody was exchanged into buffer A before use.

For probing MinE^1–31^ on the SLB, Texas Red-DHPE in the SLB recipe was reduced to 0.04 mol%. We followed the previously described protocol to induce membrane deformation, then slowly withdrew all the solution from the chamber to remove unbound proteins, and immediately applied 200 µl fresh buffer A carefully back into the chamber. A control experiment was performed in parallel with buffer in place of the MinE solution. In both the test and control samples, 10 µl Atto488 conjugated anti-MinE antibodies were added into the chamber. The chambered slides were placed in a moisture box and incubated at 4°C overnight with gentle shaking. Prior to image acquisition, the bilayer was washed by slowly withdrawing 150 µl solution and replacing with the same volume of fresh buffer A. This step was repeated five times to remove excess antibody. Samples were then ready for image acquisition.

## Supporting Information

Figure S1 **Helical wheel projections of the extreme N-terminus of MinE from 12 bacterial species.**
(TIF)

Figure S2 **Tryptophan blue shift assays of peptides with a single tryptophan substitution at A2, L3, L4, F6, F7, and L8.** A peptide with tryptophan appended to the C-terminus of MinE^1–31^ was used as a control. The blue shift at the maximal emission wavelength is indicated on top of each chart (unit: nm). cps: counts per second.
(TIF)

Figure S3 **Molecular dynamics simulation of the MinE^2-12^-membrane complex.** (**a**) Selected frames (starting model, 1 ps, 2.5 ns, 5 ns, 7.5 ns, and 10 ns) from the conformation trajectory of a 10 ns simulation. The parallel cyan lines represent the helical conformation. Horizontal blue line: membrane boundary; magenta dash line: hydrogen bond. (**b**) Diagram of the potential energy fluctuation over time. The arrow indicates the potential energy of the starting model for simulation.
(TIF)

Figure S4 **Comparison of the different membrane deformation activities of full-length MinE, MinE^1–31^, MinE^32–88^, and MacA^1–31^.** Multiple examples of Texas Red DHPE-labeled liposomes in the presence of full-length MinE (**a**), MinE^1–31^ (**b**), MinE^32–88^ (**c**), and MacA^1–31^ (**d**). Time zero was defined as the first frame acquired after the addition of protein. The scale bar indicates the diameter of a liposome.
(TIF)

Figure S5 **Mixing purified MinE^32-88^ with MinE^1-31^ was insufficient to restrict the liposome deformation activity in a confined area.** (**a**) Time sequences of liposome deformation. The scale bar indicates the diameter of a liposome. (**b**) Sedimentation assay showing MinE^32-88^ was unable to interact with MinE^1-31^ in the presence of liposomes (PE:PG:CL = 36:14:50 mol%). The statistics were obtained from 4 (reactions 1-4) or 8 (reactions 5 & 6) repeats. It should be noted that mixing MinE^1-31^ with MinE^32-88^ in buffer caused aggregation of both domains in the absence of liposomes, for unknown reasons. S, supernatant; P, pellet.
(TIF)

Figure S6 **Fluorescence recovery after photobleaching (FRAP) analysis of fluidity of the supported lipid bilayers (SLBs).** (**a**) Selected frames in a photobleaching experiment. We viewed an area of the SLBs for 45 s (10 frames, 5-s intervals) before pulling out the field stop in the light path of the microscope to define the target area, and setting the illumination power to high to cause photobleaching until a significant reduction of the fluorescence intensity occurred. The imaging conditions were then reverted back to the original settings and more images were acquired. Scale bar: 10 µm. (**b**) Kymogram of a selected area from the image sequence in (a). A micrograph on top illustrates the area selected for the kymogram. The upper row shows a kymogram prepared from the entire image sequence. The bottom row shows the fluorescence intensity map of a kymogram that was analyzed in Matlab, as described in “Materials and Methods”.
(TIF)

Figure S7 **Colocalization of MinE^1–31^ with the membrane tubules and patches.** A comparison of the stiff membrane tubules induced by MinE (**a**) and the smooth contour of the membrane tubules caused by external forces (**b**) from the SLBs. (**c**) Colocalization of MinE^1–31^ and the fluorescent membrane patches. Atto488 labeled anti-MinE antiserum was applied to the deformed SLBs to probe for MinE^1–31^. MinE^1–31^ was found around the membrane patches as enclosed circles (white arrows), arcs (yellow arrow), and partially colocalized with the membrane patches (cyan arrow). (**d**) A control for (c) in which no MinE^1–31^ was added to the sample.
(TIF)

Figure S8 **Liposome deformation activities of mutant MinE proteins (C1 and F6D) in real-time.** Five independent image sequences are presented for (**a**) C1 mutant (MinE^R10G/K11E/K12E^) and (**b**) MinE^F6D^. (**c**) A double label experiment containing Alexa Fluor 488-labeled MinE^F6D^ and Texas Red-labeled liposomes did not show significant binding of the protein to the liposome, which was in contrast to the wild-type protein in the assay.
(TIF)

Table S1 **Summary of proteins showing in vitro tubulation activity.**
(DOCX)
