# Supplementary figures and images for: The N-Terminal Amphipathic Helix of the Topological Specificity Factor MinE Is Associated with Shaping Membrane Curvature
Source: PLoS One. 2011 Jun 27;6(6):e21425. doi: 10.1371/journal.pone.0021425 (PMC3124506; doi:10.1371/journal.pone.0021425)

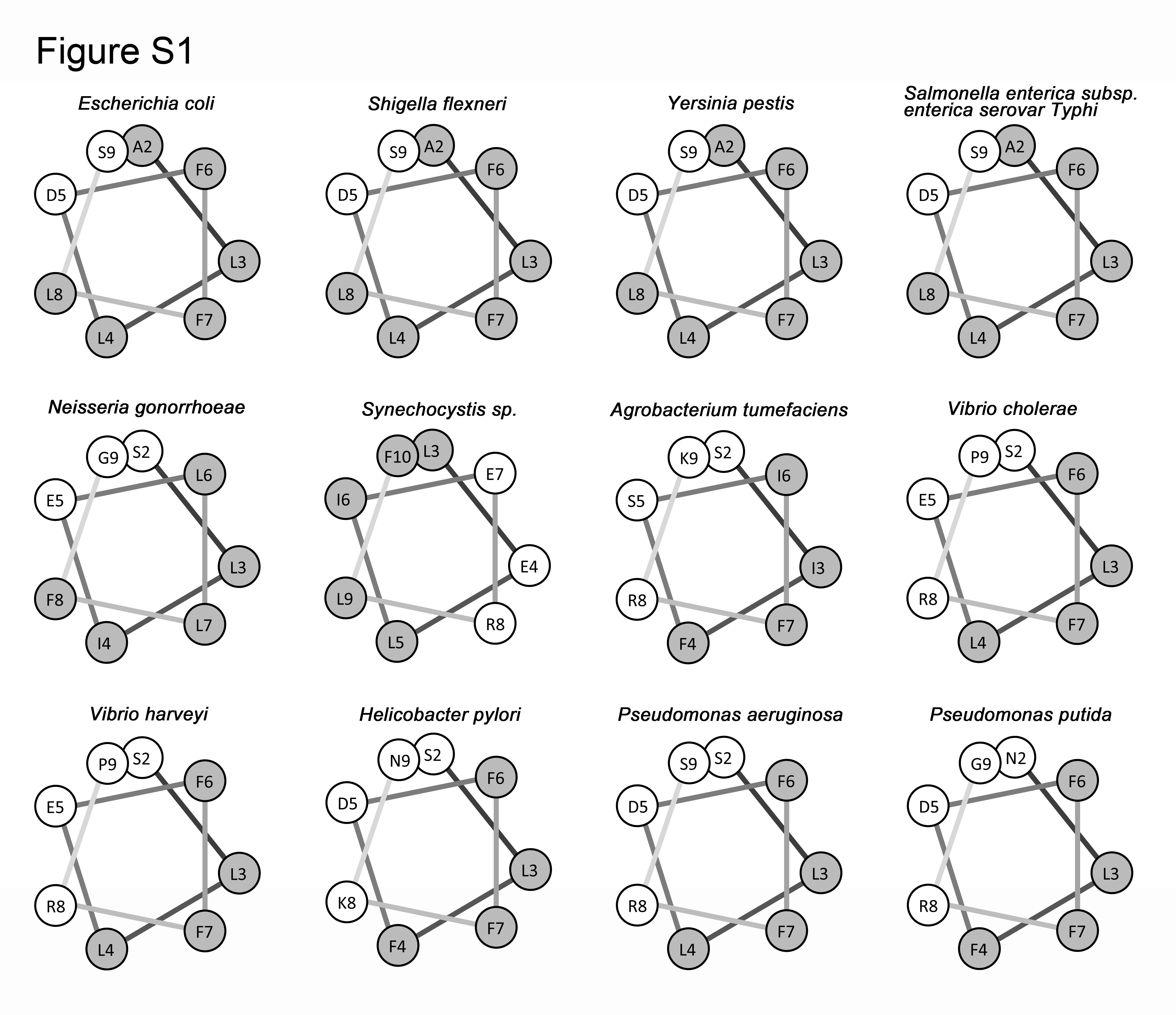

Supplement: Figure S1 — Helical wheel projections of the extreme N-terminus of MinE from 12 bacterial species. (TIF) [file pone.0021425.s001.tif]

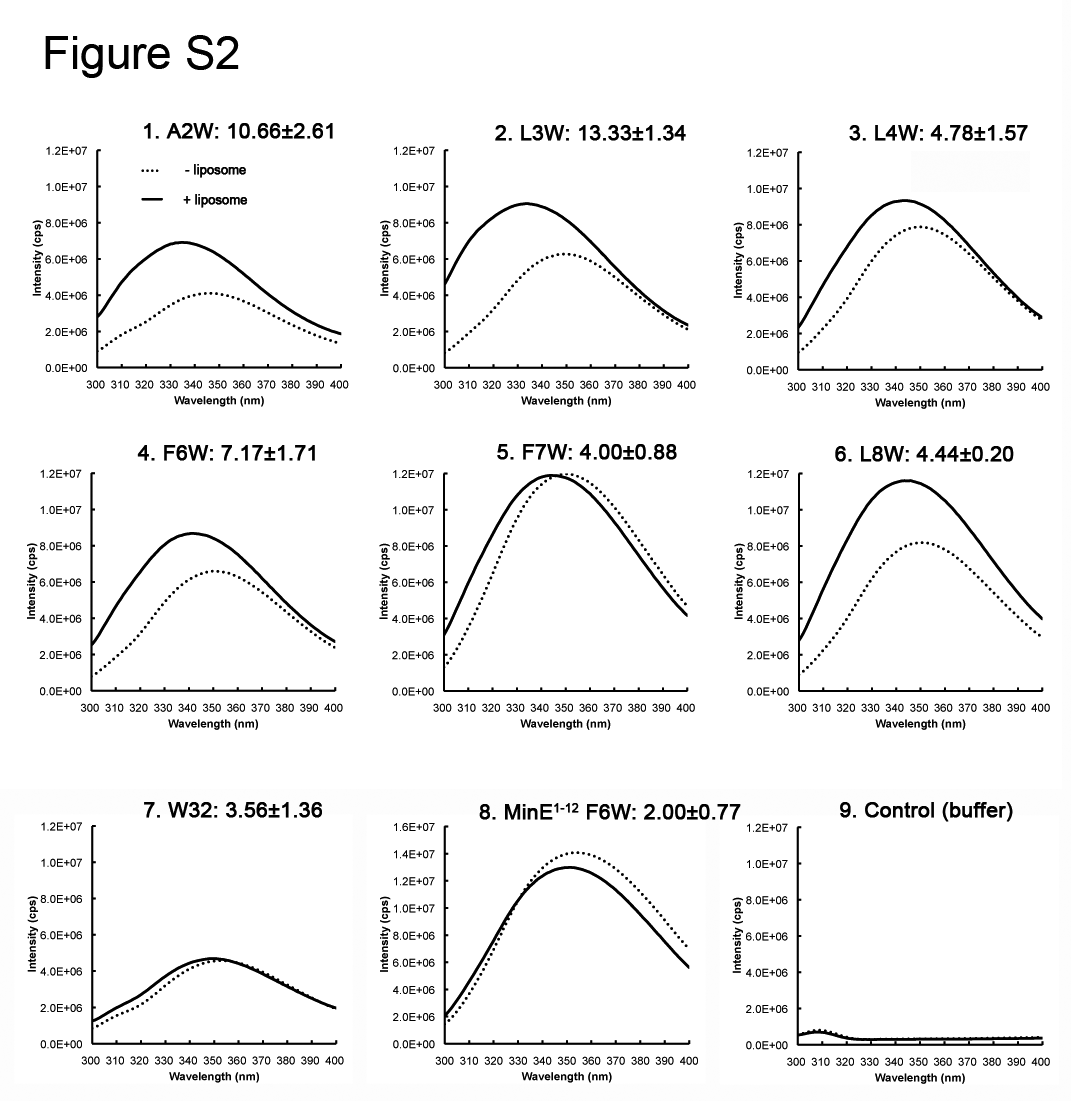

Supplement: Figure S2 — Tryptophan blue shift assays of peptides with a single tryptophan substitution at A2, L3, L4, F6, F7, and L8. A peptide with tryptophan appended to the C-terminus of MinE1–31 was used as a control. The blue shift at the maximal emission wavelength is indicated on top of each chart (unit: nm). cps: counts per second. (TIF) [file pone.0021425.s002.tif]

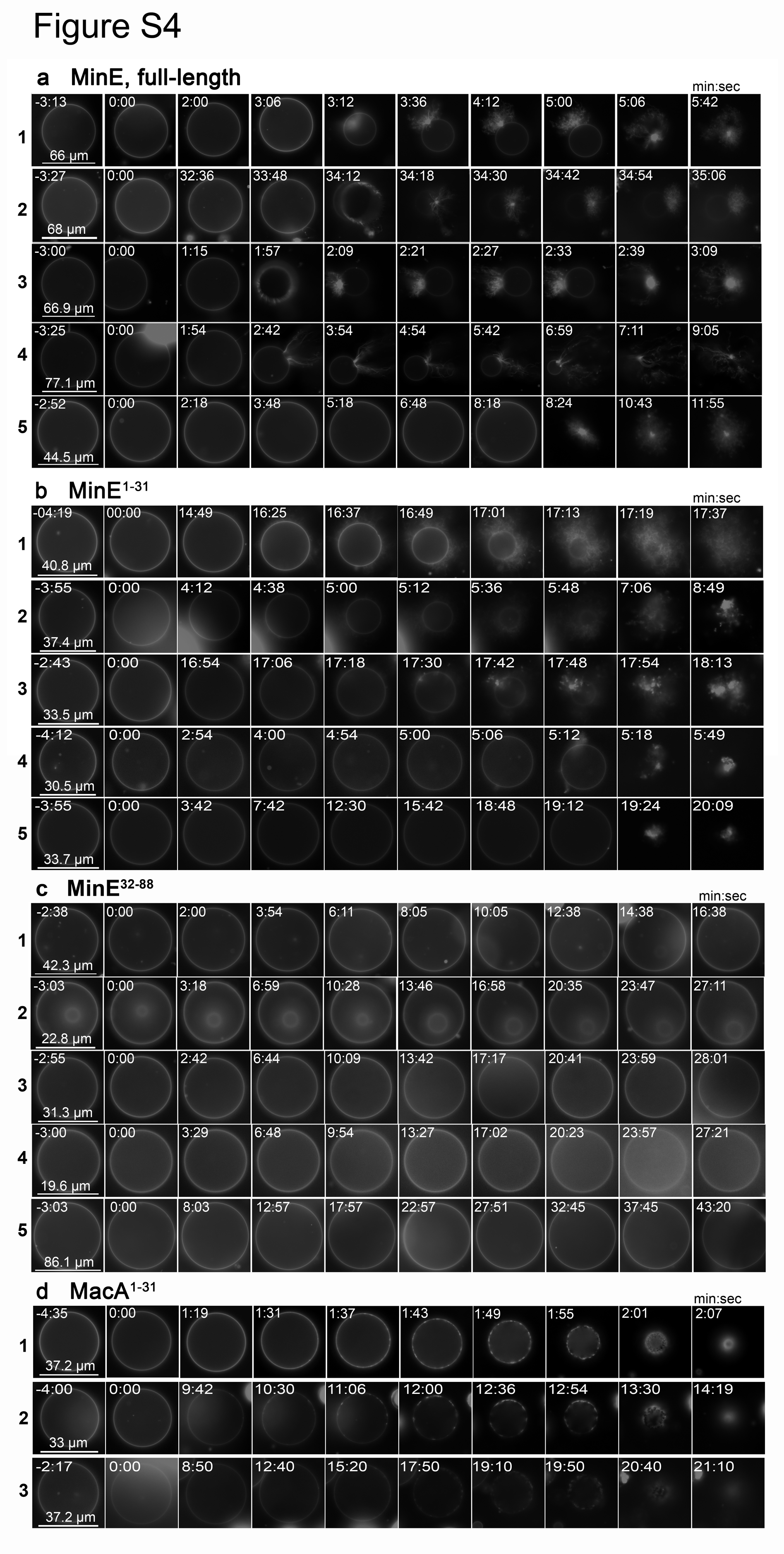

Supplement: Figure S4 — Comparison of the different membrane deformation activities of full-length MinE, MinE1–31, MinE32–88, and MacA1–31. Multiple examples of Texas Red DHPE-labeled liposomes in the presence of full-length MinE (a), MinE1–31 (b), MinE32–88 (c), and MacA1–31 (d). Time zero was defined as the first frame acquired after the addition of protein. The scale bar indicates the diameter of a liposome. (TIF) [file pone.0021425.s004.tif]

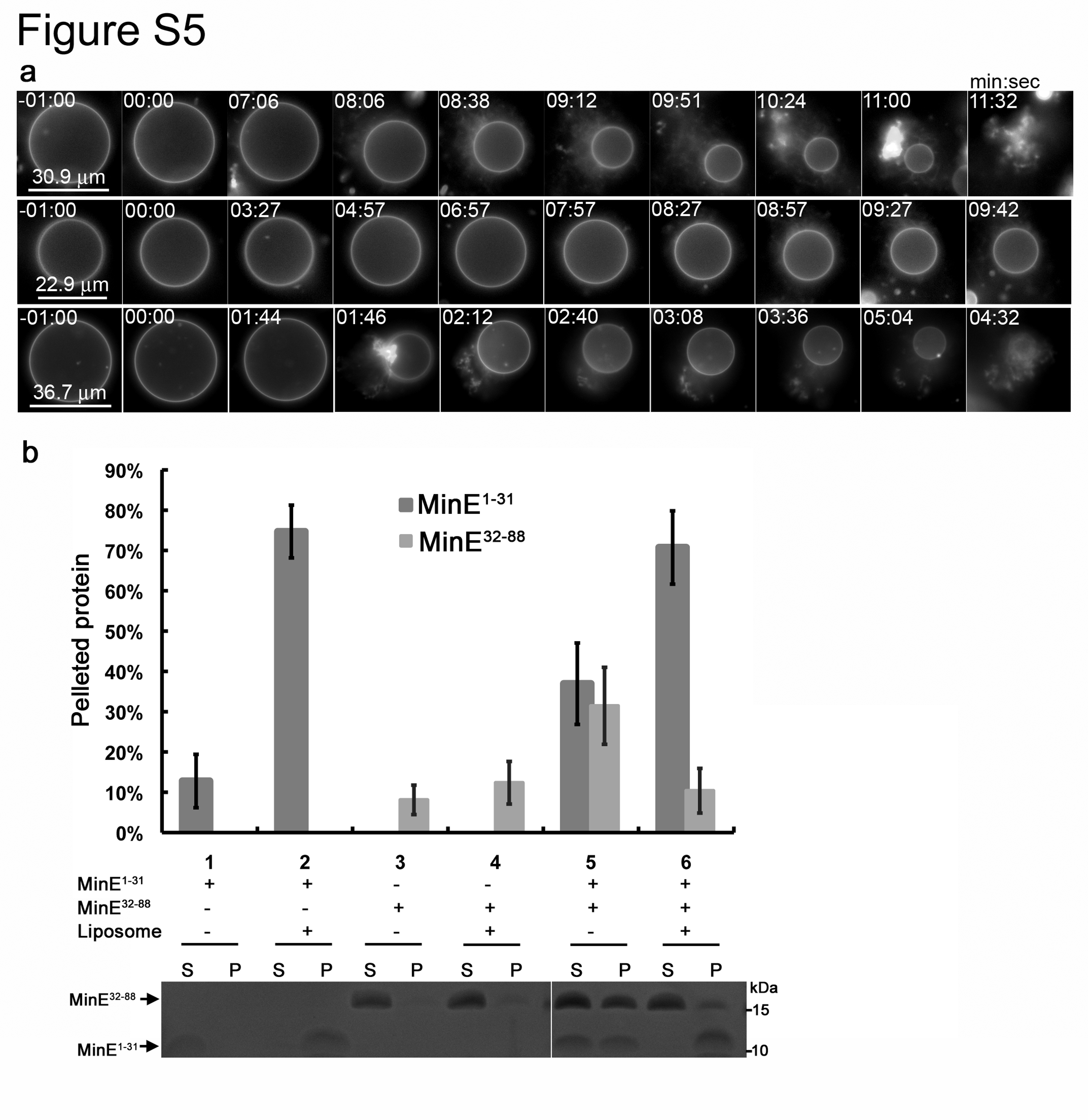

Supplement: Figure S5 — Mixing purified MinE32-88 with MinE1-31 was insufficient to restrict the liposome deformation activity in a confined area. (a) Time sequences of liposome deformation. The scale bar indicates the diameter of a liposome. (b) Sedimentation assay showing MinE32-88 was unable to interact with MinE1-31 in the presence of liposomes (PE:PG:CL = 36:14:50 mol%). The statistics were obtained from 4 (reactions 1-4) or 8 (reactions 5 & 6) repeats. It should be noted that mixing MinE1-31 with MinE32-88 in buffer caused aggregation of both domains in the absence of liposomes, for unknown reasons. S, supernatant; P, pellet. (TIF) [file pone.0021425.s005.tif]

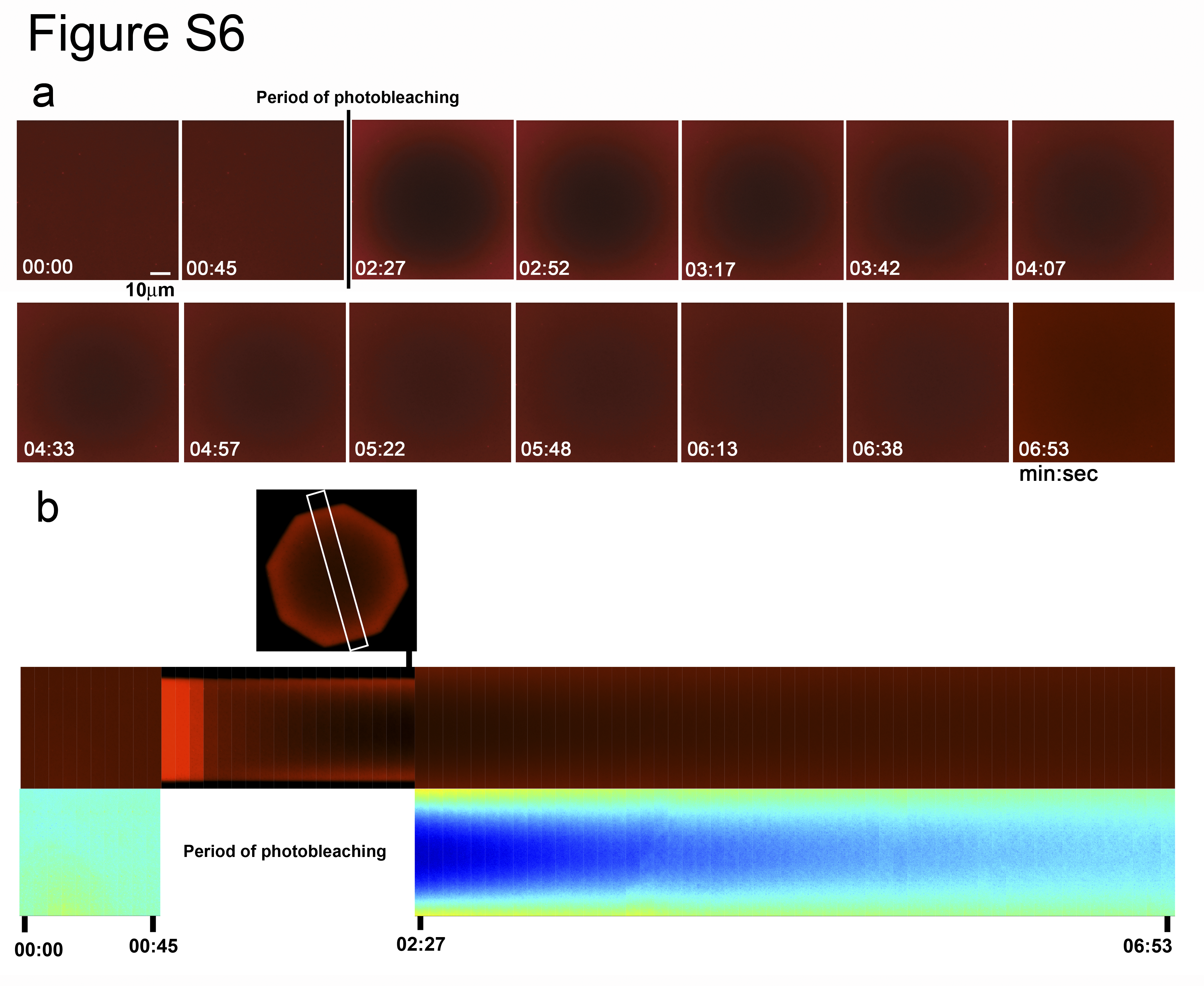

Supplement: Figure S6 — Fluorescence recovery after photobleaching (FRAP) analysis of fluidity of the supported lipid bilayers (SLBs). (a) Selected frames in a photobleaching experiment. We viewed an area of the SLBs for 45 s (10 frames, 5-s intervals) before pulling out the field stop in the light path of the microscope to define the target area, and setting the illumination power to high to cause photobleaching until a significant reduction of the fluorescence intensity occurred. The imaging conditions were then reverted back to the original settings and more images were acquired. Scale bar: 10 µm. (b) Kymogram of a selected area from the image sequence in (a). A micrograph on top illustrates the area selected for the kymogram. The upper row shows a kymogram prepared from the entire image sequence. The bottom row shows the fluorescence intensity map of a kymogram that was analyzed in Matlab, as described in “Materials and Methods”. (TIF) [file pone.0021425.s006.tif]

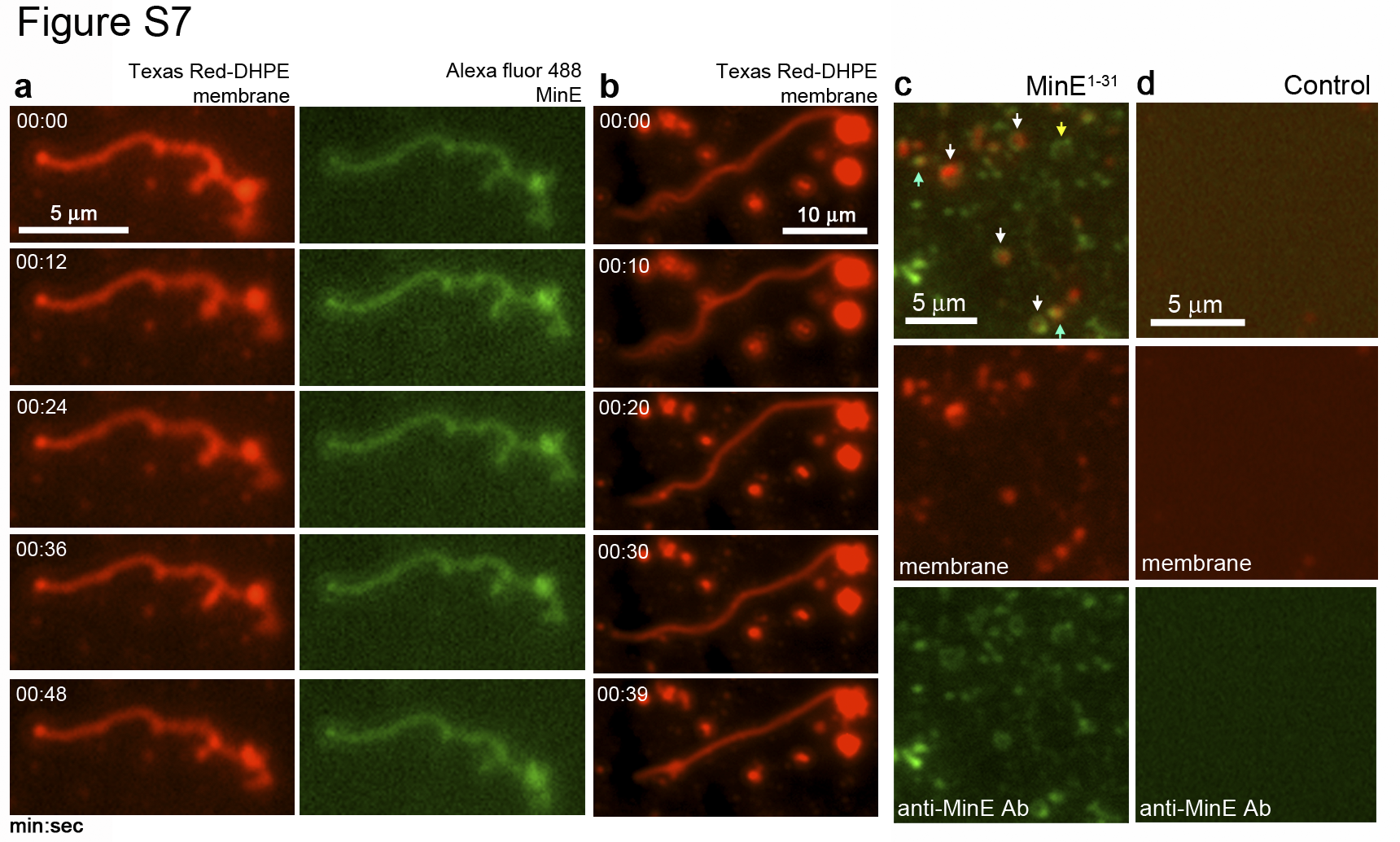

Supplement: Figure S7 — Colocalization of MinE1–31 with the membrane tubules and patches. A comparison of the stiff membrane tubules induced by MinE (a) and the smooth contour of the membrane tubules caused by external forces (b) from the SLBs. (c) Colocalization of MinE1–31 and the fluorescent membrane patches. Atto488 labeled anti-MinE antiserum was applied to the deformed SLBs to probe for MinE1–31. MinE1–31 was found around the membrane patches as enclosed circles (white arrows), arcs (yellow arrow), and partially colocalized with the membrane patches (cyan arrow). (d) A control for (c) in which no MinE1–31 was added to the sample. (TIF) [file pone.0021425.s007.tif]

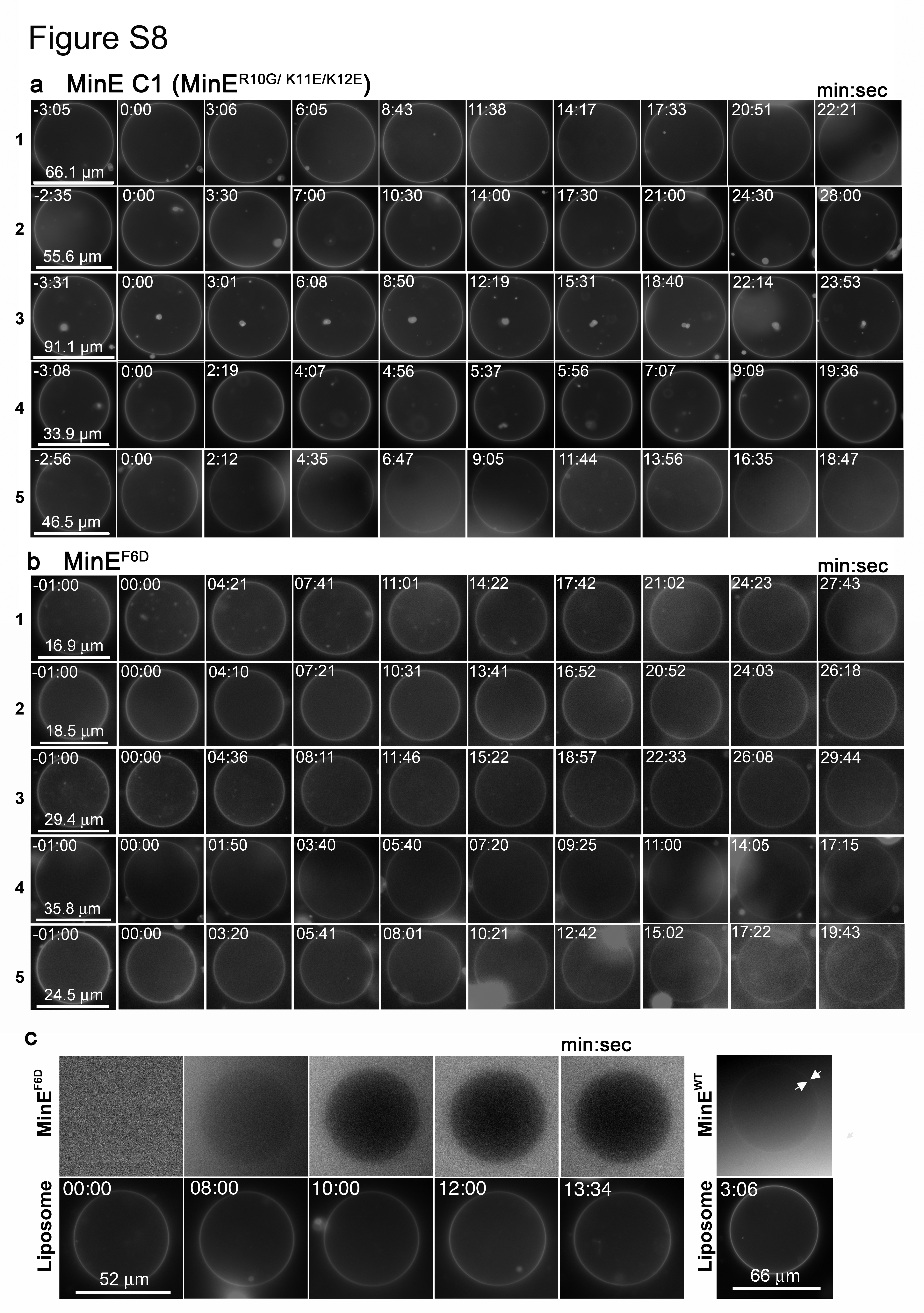

Supplement: Figure S8 — Liposome deformation activities of mutant MinE proteins (C1 and F6D) in real-time. Five independent image sequences are presented for (a) C1 mutant (MinER10G/K11E/K12E) and (b) MinEF6D. (c) A double label experiment containing Alexa Fluor 488-labeled MinEF6D and Texas Red-labeled liposomes did not show significant binding of the protein to the liposome, which was in contrast to the wild-type protein in the assay. (TIF) [file pone.0021425.s008.tif]
