# Supplementary material for: The N-Terminal Amphipathic Helix of the Topological Specificity Factor MinE Is Associated with Shaping Membrane Curvature
Source: PLoS One. 2011 Jun 27;6(6):e21425. doi: 10.1371/journal.pone.0021425 (PMC3124506; doi:10.1371/journal.pone.0021425)
Supplement: Table S1 — Summary of proteins showing in vitro tubulation activity. (DOCX) [file pone.0021425.s009.docx]

**Table S1. Summary of proteins showing *in vitro* tubulation activity.**

| **Protein** | **Function** | **Membrane-binding motif** | **Self-assembly** | **NTPase** | **Note** |
| --- | --- | --- | --- | --- | --- |
| **MinD ^[10]^** | Min oscillation | Amphipathic helix | + | ATPase | Bacterial origin |
| **MinE ^[16]^** | Min oscillation | Basic residues  Amphipathic helix | + | - | Bacterial origin |
| **BDLP ^[20]^** | Not confirmed | Hydrophobic paddle | + | GTPase | Bacterial origin |
| **Dynamin ^[40,41,42,43]^** | Clatherin-coated vesicle | Charge (PH domain) | + | GTPase | GTP regulates constriction |
| **Bar domain ^[44]^** | Clatherin-coated vesicle | Basic residues  Amphipathic helix  Protein shape | +  (F-Bar domain) | - | - |
| **ENTH domain ^[45]^** | Clatherin-coated vesicle | Amphipathic helix | - | - | - |
| **Arf ^[46,47,48,49,50]^** | COPI,  Clatherin-coated vesicle | Amphipathic helix | -  (dimerization) | GTPase | GTP induces folding of AH |
| **SarI ^[51,52]^** | COPII vesicle | Amphipathic helix | - | GTPase | GTP induces exposure of AH |
| **Septin ^[53,54]^** | Cytokinesis, exocytosis,  cell surface organization | Through interacting partner  or N-terminal charge | + (Oligomer *in vitro*,  higher order *in vivo*) | GTPase | - |
| **Synaptotagmin ^[55]^** | Synaptic vesicle fusion | Charge, hydrophobicity  (C2 domain) | - | - | Ca^2+^-dependent |
